# Studying the Humoral Response against SARS-CoV-2 in Cuban COVID-19 Recovered Patients

**DOI:** 10.1155/2024/7112940

**Published:** 2024-09-25

**Authors:** Ivette Orosa Vázquez, Marianniz Díaz, Yaima Zúñiga Rosales, Klayris Amada, Janoi Chang, Ernesto Relova Hernández, Yaima Tundidor, Hilda Roblejo Balbuena, Giselle Monzón, Bárbara Torres Rives, Enrique Noa Romero, Danay Carrillo Valdés, Irinia Valdivia Álvarez, Aurora Delahanty Fernández, Claudia Díaz, Joaquín Solozabal, Mileidys Gil, Belinda Sánchez, Gertrudis Rojas, Beatriz Marcheco, Tania Carmenate

**Affiliations:** ^1^ Center of Molecular Immunology, 15th Avenue and 216 Street, Siboney, Playa, Havana, Cuba; ^2^ National Center of Medical Genetics, 31st Avenue, N°3102 and 146 Street, Cubanacán, Playa, Havana, Cuba; ^3^ Julio Trigo Hospital, km 7½ Calzada de Bejucal, Diez de Octubre, Havana, Cuba; ^4^ Research Center of Civil Defense, José de las Lajas, San, Mayabeque, Cuba; ^5^ Centre for Immunoassays, 134st. And 25, Cubanacán, Playa, Havana, Cuba

## Abstract

Understanding the immune response generated by SARS-CoV-2 is critical for assessing efficient therapeutic protocols and gaining insights into the durability of protective immunity. The current work was aimed at studying the specific humoral responses against SARS-CoV-2 in Cuban COVID-19 convalescents. We developed suitable tools and methods based on ELISA methodology, for supporting this evaluation. Here, we describe the development of an ELISA for the quantification of anti-RBD IgG titers in a large number of samples and a similar test in the presence of NH_4_SCN as chaotropic agent for estimating the RBD specific antibody avidity. Additionally, a simple and rapid ELISA based on antibody-mediated blockage of the binding RBD-ACE2 was implemented for detecting, as a surrogate of conventional test, the levels of anti-RBD inhibitory antibodies in convalescent sera. In a cohort of 273 unvaccinated convalescents, we identified higher anti-RBD IgG titer (1 : 1,330, *p*  < 0.0001) and higher levels of inhibitory antibodies blocking RBD-ACE2 binding (1 : 216, *p*  < 0.05) among those who had recovered from severe illness. Our results suggest that disease severity, and not demographic features such as age, sex, and skin color, is the main determinant of the magnitude and neutralizing ability of the anti-RBD antibody response. An additional paired longitudinal assessment in 14 symptomatic convalescents revealed a decline in the antiviral antibody response and the persistence of neutralizing antibodies for at least 4 months after the onset of symptoms. Overall, SARS-CoV-2 infection elicits different levels of antibody response according to disease severity that declines over time and can be monitored using our homemade serological assays.

## 1. Introduction

At the end of 2019, the novel betacoronavirus severe acute respiratory syndrome coronavirus 2 (SARS-CoV-2) was detected in China, and to date, it has infected millions of people worldwide [1]. Consequently, the coronavirus disease 2019 (COVID-19) caused by SARS-CoV-2, resulted in a pandemic and a public health crisis [2]. The clinical manifestations of COVID-19 range from asymptomatic to mild, moderate, severe, and fatal disease [3]. The genome of SARS-CoV-2 encodes four major structural proteins: spike protein (S), nucleoprotein (N), membrane protein (M), and envelope protein (E) [4]. The receptor-binding domain (RBD), contained in the S1 subunit of the S protein, mediates viral entry by binding to the angiotensin converting enzyme-2 (ACE2) receptor on the human host cell [5]. SARS-CoV-2 infection elicits both humoral and cellular immunity which play crucial roles in controlling and resolving viral infection and COVID-19. Indeed, strong SARS-CoV-2 specific CD4+ or CD8+ T cell responses were associated with low disease severity [6]. Studies have indicated that depletion of CD8+ T cells partially abrogated the protection of convalescent macaques against rechallenge with SARS-CoV-2 [7]. Beside the cellular response, neutralizing antibodies against SARS-CoV-2, which interfere with the entry of the virus into target cells, have been considered crucial for protection against virus infection [8]. Many neutralizing antibodies target the RBD [9], while others with nonneutralizing activity are developed against N, to a lesser extent, against M protein [10]. Therefore, several serological tests have been developed and validated to study the antiviral response during the acute and convalescent phases of COVID-19, where the main antigenic targets used are RBD, S, and N proteins [11].

Thus, by considering the relevance of the antibody response in the resolution of COVID-19, we analyzed the humoral response against SARS-CoV-2 in a cohort of 273 Cuban recovered patients that were stratified by COVID-19 disease severity. We also explored the dynamics of antibody response to infection in a short cohort of convalescents.

## 2. Materials and Methods

### 2.1. Generation of Stable Clones Producing RBD-mFc

The gene coding for Wuhan-Hu-1 RBD, optimized for mammalian cell expression and synthesized by EurofinsTM, was subsequently cloned into the intermediate vector pCMX/mFc using the restriction sites BssHII/NotI (New England Biolabs, USA). The whole expression cassette, comprising CMV promoter and the genes coding for a mouse IgG heavy chain signal peptide, the RBD sequence (aa 328-533) and a mouse IgG2a Fc region, was amplified by PCR and cloned into the lentiviral vector pL6WBlast (Centro de Ingeniería Genética y Biotecnología, Cuba) using XhoI/EcoRV restriction sites (New England Biolabs, USA). Sequence correctness was verified by Microsynth Seqlab, Germany. Lentiviral particles were assembled by adherent HEK-293T cells co-transfected with this genetic construct and the auxiliary plasmids pLPI, pLPII, and pLP/VSV-G (Invitrogen, USA). Viral particles were precipitated from supernatant with polyethylene glycol, and titrated by ELISA with DAVIH Ag p24 ELISA kit (LISIDA, Cuba). HEK-293 cells were grown in individual wells of 96-well cell culture plates containing DMEMF12 medium (Life Technologies, USA) supplemented with 5% (v : v) heat-inactivated fetal bovine serum, and infected at 800 multiplicity of infection (MOI) during 8 hr in DEMEMF12. Viral transduction was repeated 24 and 48 hr later in the same conditions. Transduced cells were diluted and expanded to 96-well plates in the presence of 2 *µ*g/mL of the selection drug blasticidin (Gibco, USA). Fc-fusion protein in the supernatants was detected using a sandwich ELISA, based on polyvinyl chloride microtitration plates coated with an anti-mouse IgG antibody and a second anti-mouse IgG antibody conjugated with horseradish peroxidase (HRP; #A2554, Sigma, USA). Oligoclonal cell populations secreting the highest levels of fusion protein were cloned by limiting dilution, the clones were screened by ELISA. The highest producer clones were expanded and adapted to grow in suspension in the proprietary serum-free medium MB06. RBD-mFc was purified from cell culture supernatant through protein A affinity chromatography (Cytiva, USA).

### 2.2. Characterization of RBD-mFc Fusion Protein

Purity and integrity of the purified fusion protein was shown by SDS/PAGE under reducing conditions on a 7.5% gel. The aggregation status of the purified fusion protein was analyzed by size exclusion chromatography in a TSK gel G3000SWXL column (Tosoh Bioscience, Japan). The initial assessment of antigenicity and biological activity was carried out by ELISA on polyvinyl chloride microtitration plates coated either the recombinant human antibody SCV2-RBD-100 m (eEnzyme, USA) or a fusion protein comprising ACE2 extracellular domain fused to human IgG1 Fc region, respectively. Coating molecules were diluted at 5 *µ*g/mL in phosphate-buffered saline (PBS) and incubated on plates overnight at 4°C. After discarding coating solutions, plates were blocked with skim powder milk at 4% (w : v) in PBS (M-PBS) for 30 min at room temperature (RT). RBD-mFc was diluted in M-PBS and incubated on coated/blocked plates 1 hr at RT. After washing with PBS containing 0.01% (v : v) Tween 20 (PBS-T), plates were incubated with an anti-mouse IgG antibody conjugated to HRP, properly diluted in M-PBS. Plates were washed and peroxidase substrate solution (ortho-phenylenediamine (Sigma, USA) at 0.5 mg/mL and 0.015% (v : v) hydrogen peroxide in 0.1 mol/L citrate-phosphate buffer, pH 5) was added. After 15 min, the reaction was stopped with 10% (v : v) sulfuric acid. The optical density (OD) at 490 nm were measured in a microplate reader (Organon Teknica, Salzburg, Austria).

### 2.3. Study Design and Participants

We conducted two cross-sectional studies in convalescents from confirmed SARS-CoV-2 infection by the real-time polymerase chain reaction (RT-PCR), who were recruited during the first and second pandemic waves in Cuba. The enrolled participants included subjects who met all the inclusion and exclusion criteria. Inclusion criteria were subjects of both sexes, over 1 year of age, who were willing and able to participate in the study and had viral clearance confirmed through one negative result of the RT-PCR for SARS-CoV-2. Exclusion criteria included deceased, individuals who were not Cuban residents, and individuals with physical and/or mental limitations and the absence of relatives to respond to the clinical interview.

The first cross-sectional study included 273 confirmed SARS-CoV-2 infection cases in all provinces of Cuba between March and June, 2020. For the second study, we included 14 COVID-19 convalescents, who reported with symptoms between May and October, 2020. We reviewed the clinical records of patients, using an analysis guide, and a face-to-face interview was mainly based on sociodemographic characteristics (age, biological sex, territory of residence, skin color, marital status, occupation, and concluded educational level), clinical (comorbidities), and epidemiological data (date of SARS-CoV-2 infection diagnose). Disease severity was defined as asymptomatic (confirmed cases that never had any symptoms of COVID-19), mild/moderate (symptomatic cases with nonsevere pneumonia or not, and who have saturation of oxygen SpO2 ≥90% on room air), and severe (individuals who require hospitalization in the intensive care unit, defined in the Cuban protocol, with growing dyspnea and who have SpO2 <90% in room air) [12]. Furthermore, to test the specificity of the implemented assays, serum samples were obtained from 79 individuals who were close contact of SARS-CoV-2 infected patients (cohabitants) but had tested negative for the SARS-CoV-2 virus by RT-PCR during epidemiological monitoring. Additionally, 25 sera collected before COVID-19 pandemic (2017–2018) were used as controls for the development of our ELISAs. Sample size was not previously computed but was limited by the number of available subjects and samples as well as the available testing capacity; a consecutive sampling method was used.

### 2.4. ELISAs to Assess Anti-RBD Humoral Response

**Figure 1:**
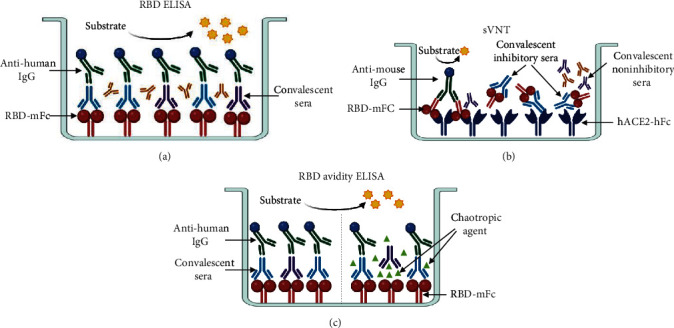
Schematic representation of three different types of ELISA for characterizing the anti-RBD humoral response elicited by SARS-CoV-2 infection. (a) RBD ELISA set up for detecting recognition and binding of convalescent IgG antibodies to RBD. (b) Surrogate virus neutralization test (sVNT) design for evaluating the inhibitory capacity of anti-RBD IgG antibodies in blocking the binding of RBD-ACE2. (c) RBD avidity ELISA set up for estimating the IgG avidity of IgG antibodies against RBD.

We developed a set of serological assays for characterizing the magnitude, inhibition capacity, and avidity of RBD-specific antibody response. Our first assessment was designed for quantifying anti-RBD IgG titer by ELISA (RBD ELISA), which involves coating ELISA plates with RBD-mFc, incubate with convalescent sera, and detect the bound antibodies with a peroxidase conjugated anti-human IgG monoclonal antibody (Figure 1(a)). The second strategy was focused on detecting inhibitory antibodies able to block RBD-ACE2 binding, by a surrogate virus neutralization test (sVNT), as alternative tool to detect neutralizing antibodies. This strategy utilizes ELISA plates coated with hACE2-hFc, then employing a mixture of convalescent sera and RBD-mFc, and detecting the bound RBD (Figure 1(b)). The last strategy was designed for estimating the anti-RBD IgG avidity index (RBD Avidity ELISA). This ELISA is similar to our first strategy (RBD ELISA), but after the sera incubation step, we add chaotropic agent (NH_4_SCN) to disrupt the binding between RBD and antibodies (Figure 1(c)). The details for each assay are described below.

### 2.5. ELISA to Assess the Anti-RBD Specific IgG Titers (RBD ELISA)

96-well plates (Maxisorp, Thermo Scientific, USA) were coated with 5 *μ*g/mL of RBD-mFc and incubated at 37°C for 2 hr. Plates were washed with PBS-T and blocked (PBS-T 4% milk) for 30 min at RT. Serum samples were serially diluted in sample buffer (PBS-T and 2% milk) in duplicate, added to plates, and incubated for 2 hr at RT (starting at 1 : 100). After washing with PBS-T, plates were incubated with peroxidase conjugated anti-human IgG monoclonal antibody diluted at 1 : 5,000 (# A0170, Sigma, USA) in sample buffer for 1 hr at RT. Plates were washed and peroxidase substrate solution was added. After 15 min, the reaction was stopped with 10% (v : v) sulfuric acid. The OD at 490 nm were measured in a microplate reader. Antibody titers were considered as the inverse of the highest serum dilution giving OD values that are fourfold the value of the negative control serum.

### 2.6. ELISA to Assess the Inhibition of Binding of RBD-mFc to hACE2-hFc by Antibodies (sVNT)

96-well plates were coated with 5 *μ*g/mL of hACE2-hFc and incubated overnight at 4°C. Plates were washed with PBS-T and blocked (PBS-T 2% milk) for 30 min at RT. Serum samples were serially diluted in duplicate in sample buffer (PBS-T 0.2% milk) starting at 1 : 25 and incubated with RBD-mFc (at a final concentration of 20 ng/mL) for 1 hr at 37°C. Blocked plates were washed and incubated with the premixed serum and RBD-mFc for 2 hr at RT. Next, alkaline phosphatase conjugated anti-mouse IgG antibody (1 : 1,000, #A9316, Sigma, USA) was added and incubated for 1 hr at 37°C. Finally, the substrate solution *p*-nitrophenylphosphate (Sigma, USA; 1 mg/mL in diethanolamine buffer (pH 9.8)) was added, and plates were incubated at RT for 30 min, protected from the light. The OD at 405 nm was measured using a microwell system reader.

Inhibition was calculated and expressed as a percentage according to the following formula: Inhibition (%) *=* [1−(OD_405 nm_ sample/OD_405 nm_ maximal recognition)] × 100. Maximal recognition corresponds to RBD-mFc (20 ng/mL). The molecular virus neutralizing titer was considered as the titer required to obtain 50% inhibition of RBD-ACE2 binding. Half-maximal inhibitory dilution (ID50) was calculated for each serum sample using sVNT assay. Dilutions were transformed at a logarithmic scale and the data were adjusted to a nonlinear function using the equation: log (inhibitor) vs. normalized response with a variable slope. The ID50 was determined using GraphPad Prism software (California, USA).

### 2.7. ELISA to Estimate the Anti-RBD IgG Avidity Index (RBD Avidity ELISA)

For the RBD specific IgG avidity assay, the coating and blocking steps were performed as described above for anti-RBD ELISA. Afterwards, the plates were incubated with serum samples diluted at 1 : 100 in sample buffer (PBS-T and 0.2% milk) in duplicate for 2 hr. Plates were washed with PBS-T and incubated for 15 min with twofold serial dilutions of NH_4_SCN (starting at 4 M; Merck, Germany). After four wash steps, plates were incubated with peroxidase conjugated anti-human IgG monoclonal antibody at 1 : 5,000 (# A0170, Sigma, USA) and incubated for 1 hr at RT. Then, plates were washed and peroxidase substrate solution was added. After 15 min, the reaction was stopped with 10% (v : v) sulfuric acid. The OD at 490 nm were measured in a microplate reader. The molarity of NH_4_SCN reducing 50% of the bound antibodies was considered as the avidity index. Every sample was tested twice by independent analyst and the average is presented.

### 2.8. Statistical Analysis

GraphPad Prism V.7.04. software was used to construct all graphics and to calculate ID50 and avidity index for sera tested in inhibition or avidity assays. Statistically significant associations were explored with appropriate parametric or nonparametric tests: Kruskal–Wallis test, adjusted for multiple comparisons by Dunn's test to compare the anti-RBD titers and ID50 of more than two participant panels or severities groups; Mann–Whitney test, to compare the anti-RBD titers and ID50 between only two groups whose data sets were not paired; and one way ANOVA test adjusted for multiple comparisons by Holm–Sidak's test to compare avidity index of anti-RBD IgG between severities. Spearman correlation tests were used to determine the correlation between UMELISA SARS-CoV-2 anti-RBD test and RBD ELISA as well as between conventional virus neutralization test (cVNT) and sVNT. Spearman correlation coefficient greater than 0.7 indicates a strong positive correlation between variables [13]. Statistical significances are presented in the figures as asterisks ( ^*∗*^*p*  < 0.05,  ^*∗∗*^*p*  < 0.005,  ^*∗∗∗*^*p*  < 0.0005, and  ^*∗∗∗∗*^*p*  < 0.0001).

## 3. Results

### 3.1. Description of COVID-19 Recovered Patient Cohort

Specific immune characterization was performed in 273 participants, stratified in asymptomatic (*n* = 79), mild and moderate (*n* = 131), or severe (*n* = 63) acute infection as designated by the Guidelines for Clinical Management of COVID-19 of May 27, 2020 [12]. Convalescents had a median age of 49 years (range: 1–96 years), and 54% were female (*n* = 149). Individuals were recruited from multiple sites throughout Cuba during the first COVID-19 outbreak (March–June, 2020), and the average of days after they were confirmed negativity of SARS-CoV-2 by RT-PCR was 65 days (interquartile range: 56–77).

Additionally, paired samples from 14 individuals who were infected during the second pandemic wave (May–June, 2020) were studied to assess the dynamics of the RBD-specific humoral response. The first serum samples were collected at the end of the acute phase, between 3 and 17 days (t1), and the second ones in the late convalescent phase, between 60 and 120 days (t2), calculated from the day postonset of symptoms (POS). The clinical and demographic characteristics of the convalescents are summarized in the Table 1. Furthermore, serum samples from 25 subjects, collected between 2017 and 2018 (pre-COVID-19 panel) and from 79 cohabitants of the above referred COVID-19 recovered patients were included in the serological study.

### 3.2. Recombinant RBD-mFc Fusion Protein is Antigenically Functional and Biologically Active

**Figure 2:**
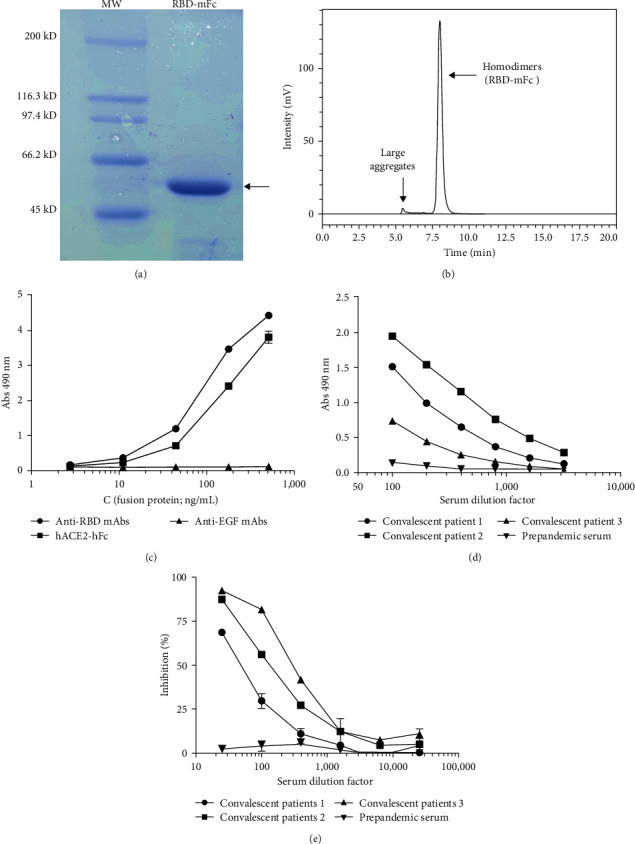
Characterization of a recombinant RBD version fused to mouse IgG2a Fc (RBD-mFc). (a) The fusion protein produced by HEK-293 cells upon stable lentiviral transduction, and purified by Protein A affinity chromatography, was analyzed by SDS/PAGE in a 7.5% gel under reducing conditions. The arrow indicates the band corresponding to pure RBD-mFc. Molecular weight ladder (MW) was included as reference. (b) The aggregation status of RBD-mFc was studied by size exclusion chromatography in a TSK gel G3000SWXL column. Elution profile shows a major peak corresponding to nonaggregated Fc-mediated homodimers and minor amounts of large aggregates. (c) Antigenicity and biological activity of RBD-mFc evaluated by ELISA on microtitration plates coated with a recombinant anti-RBD monoclonal antibody (SCV2) or the recombinant fusion protein comprising the extracellular domain of human ACE2 fused to a human IgG1 Fc domain (hACE2-hFc). The humanized R3 antibody recognizing human EGF receptor was used as an unrelated coating protein to assess nonspecific background levels. Bound RBD-mFc was detected with an anti-mouse IgG antibody conjugated to horseradish peroxidase (HRP). (d) Sera of COVID-19 convalescent patients were incubated on plates coated with RBD-mFc. Bound human antibodies were detected with an anti-human IgG mAb conjugated to HRP. (e) Binding of RBD-mFc to immobilized hACE2-hFc was inhibited by sera of COVID-19 convalescent patients. Prepandemic serum was used as negative control in the latter experiments.

SARS-CoV-2S protein fragment 328-533 (comprising the minimal RBD [14] plus short extensions at both N-and C termini and fused to a mouse IgG2a Fc domain) was successfully produced by HEK-293 host cells upon stable lentiviral transduction. More than 270 mg of purified protein (RBD-mFc; Figure 2(a)) were obtained from each liter of cell culture supernatant, which was enough for the intended analytical applications. The product was predominantly obtained (> 96%) as a homogeneous homodimer due to the presence of a self-dimerizing Fc moiety, with only minor amounts of large aggregates (Figure 2(b)).

Recognition of RBD-mFc by SCV2-RBD recombinant neutralizing monoclonal antibody, comprising variable regions isolated from immortalized human COVID-19 patient B cells (Figure 2(c)), and by a collection of sera from COVID-19 convalescent patients (Figure 2(d)) proved that RBD-mFc recombinant protein keeps the antigenicity of its natural counterpart in the virus. Furthermore, RBD-mFc was shown to be biologically active, though its interaction with another recombinant protein containing the extracellular domain of ACE2 receptor (hACE2-hFc; Figure 2(c)). Such interaction was inhibited by sera of SARS-CoV-2 infected individuals in a dose-dependent manner, but not by prepandemic serum (Figure 2(e)), further confirming its specificity. This result was the starting point for the development of a surrogate test to predict the ability of sera antibodies to inhibit virus binding to host cell receptors in a high throughput fashion.

### 3.3. Developed Immunochemical Assays Based on Recombinant RBD and ACE2 Proteins Show High Specificity and Good Correlation with Standard Assays

**Figure 3:**
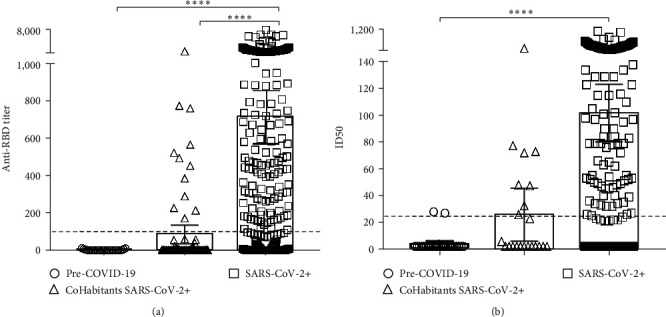
Development of ELISA tests to characterize humoral response to SARS-CoV-2 RBD. (a) Anti-RBD antibody titers were detected by RBD ELISA. Sera from COVID-19 convalescent patients (*n* = 273; SARS-CoV-2 +), prepandemic donors (*n* = 25; pre-COVID-19), and cohabitants with SARS-CoV-2+ patients (*n* = 79; cohabitants SARS-CoV-2+) were incubated on plates coated with the RBD-mFc and were detected with an anti-human IgG mAb conjugated to horseradish peroxidase (HRP). Anti-RBD IgG titer was considered to be the inverse of the highest serum dilution giving optical dilution (OD) values that were at least fourfold the value of the negative control serum. (b) Inhibition of binding of RBD-mFc to recombinant protein comprising human ACE2 extracellular domain fused to human IgG1 Fc (hACE2-hFc) by antibodies. Binding inhibition was assessed after preincubation of RBD-mFc with sera samples. Half-maximal inhibitory dilution (ID50) for every sample in each assay was determined after fitting the data to sigmoidal inhibition curves. The *p* values were calculated by the nonparametric Kruskal–Wallis test, where  ^*∗∗∗∗*^*p*  < 0.0001, adjusted for multiple comparisons by Dunn's test.

**Figure 4:**
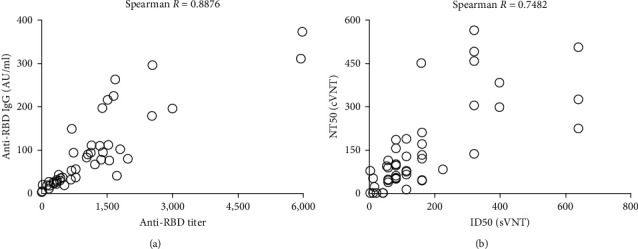
Correlation of homemade ELISAs with external validated serological tests. Spearman correlation was used to determine the association between (a) anti-RBD titer obtained by RBD ELISA and anti-RBD IgG concentration tested by validated UMELISA SARS-CoV-2 ANTI-RBD test and between (b) half-maximal neutralization dilution (NT50) obtained by conventional virus neutralization test (cVNT) and half-maximal inhibitory dilution (ID50) detected by surrogate virus neutralization test (sVNT). A significant correlation was observed (*p*  < 0.0001).

Following the evaluation of a small number of samples by the two homemade ELISAs described above, we decided to test the specificity and accuracy of the assays. The specificity of the ELISA for antibody titer detection was tested by stratifying the samples into three groups: a COVID-19 convalescent panel (SARS-CoV-2 +; *n* = 273), a prepandemic negative control group (pre-COVID-19; *n* = 25), and a panel of cohabitants with a known COVID-19 case (cohabitants SARS-CoV-2+; *n* = 79). Sera were considered positive if the 1 : 100 dilution resulted in an OD value above the assay cutoff (fourfold background). The RBD ELISA detected anti-RBD IgG titers and clearly discriminated between the study groups, showing high specificity (Figure 3(a)). Sera from COVID-19 convalescents showed higher RBD-binding antibody titers (mean anti-RBD IgG titer 1 : 713; [95% CI, 1 : 568 to 1 : 857]) compared to the prepandemic panel, where no specific IgG was detected (<1 : 100, cutoff). Sera from 79 cohabitants of COVID-19 convalescents with negative RT-PCR for SARS-CoV-2 virus during epidemiological surveillance were also analyzed. Significantly lower anti-RBD antibody titers were observed in the cohabitant cohort compared with convalescents. Although the ELISA was not fully validated, it was compared with the Cuban UMELISA SARS-CoV-2 anti-RBD test manufactured by the immunoassay center, a commercially available and validated method, and as shown in Figure 4(a), the RBD ELISA had a strong positive overall correlation with this assay (*R* = 0.8876, *p* < 0.0001).

The ability of convalescent antibodies to inhibit the binding of RBD to the ACE2 receptor has been widely evaluated as a surrogate for antibody neutralizing ability [15]. In contrast to the cVNT, we developed a rapid (8–10 hr) sVNT to detect inhibitory antibodies without the need to use infectious virus or grow cells. The test was validated following International Council for Harmonization (ICH) Q2 (R1) recommendations for validation of analytical procedures. General linear ANOVA was performed and the coefficients of variation (CVs) for repeatability (3.4%) and reproducibility (10.6%) were perfectly in line with the expected variability. The specificity of the assay was also high, with RBD-ACE2 binding specifically blocked by sera from the COVID-19 convalescent panel (mean inhibitory IgG titer 1 : 109; 95% CI, 1 : 86 to 1 : 131; Figure 3(b)). In contrast, 92% of the pre-COVID-19 samples (23/25) had inhibitory capacity below the cutoff index value (1 : 25), similar to the anti-RBD IgG results.

We randomly selected a subpanel of 47 sera with varying levels of anti-RBD IgG to be analyzed in parallel with the current gold standard cVNT assay. The number of samples tested was limited by the availability of the neutralization assays. There was a good correlation between the levels of RBD-ACE2 inhibitory antibody titers and neutralizing antibody titers detected by the current gold standard cVNT, *R* = 0.7842 and *p*  < 0.0001 (Figure 4(b)). Although the absolute values of the variables studied were dependent on the assay format, there was a positive correlation between the results of both pairs of experiments, confirming the usefulness of both homemade assays for the detection of total and inhibitory anti-RBD antibodies.

### 3.4. The Magnitude and Functionality of RBD-Specific Antibody Response Depend on Disease Severity

**Figure 5:**
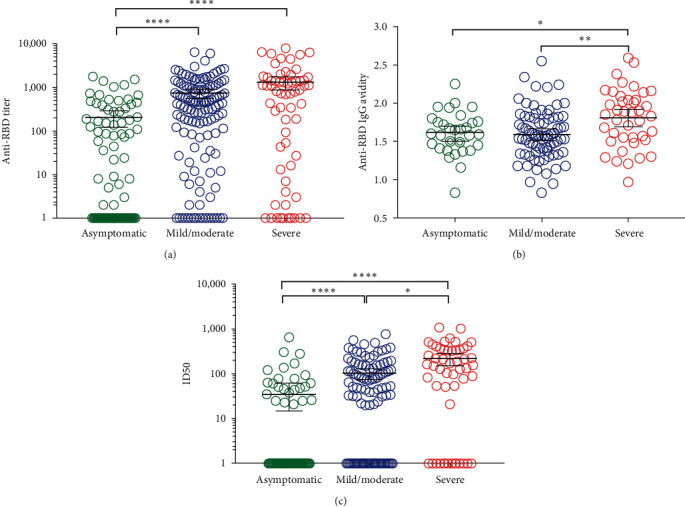
Impact of disease severity of COVID-19 recovered patients on the magnitude and quality of the anti-RBD antibody response. Sera of convalescents were stratified into three groups according to the clinical course of the disease: asymptomatic, mild/moderate, and severe. (a) Anti-RBD IgG titer was determined by RBD ELISA. Serum samples were incubated on plates coated with the RBD-mFc and were detected with an anti-human IgG mAb conjugated to horseradish peroxidase (HRP). (b) The avidity index for each sample was estimated by the incubation of sera on plates coated with the RBD-mFc, followed by the addition of NH_4_SCN. Bound human polyclonal antisera were detected with an anti-human mAb conjugated to HRP. The avidity index was considered as the molarity of NH_4_SCN reducing 50% of the bound antibodies. (c) Half-maximal inhibitory dilution (ID50) was determined by surrogate virus neutralization test (sVNT). Inhibition of binding of RBD-mFc to hACE2-hFc was assessed after preincubation of RBD-mFc with sera samples. (a) and (b) The *p* values were calculated by the nonparametric Kruskal–Wallis test, where  ^*∗∗∗∗*^*p*  < 0.0001 and  ^*∗*^*p*  < 0.05, adjusted for multiple comparisons by Dunn's test. (c) The *p* values were calculated by the one way ANOVA test, where  ^*∗*^*p*  < 0.05 and  ^*∗∗*^*p*  < 0.005, adjusted for multiple comparisons by Holm–Sidak's test.

To explore the potential association between clinical features of COVID-19 convalescents and the RBD-specific antibody response, we stratified convalescent samples into three groups based on symptoms severity: asymptomatic, mild/moderate, and severe. The mean anti-RBD antibody titer gradually increased with clinical severity: mean IgG titers _asymptomatic_, 1 : 203 (95% CI, 1 : 121 to 1 : 285) < mean IgG titers _mild/moderate_, 1 : 723 (95% CI, 1 : 547 to 1 : 900) < mean IgG titers _severe_, 1 : 1,330 (95% CI, 1 : 867 to 1 : 1794; Figure 5(a)). A similar methodology to RBD ELISA, but adding a chaotropic agent (NH_4_SCN) to elute antibodies that bind weakly to the RBD was used to estimate the avidity of antibodies in 123 samples with anti-RBD IgG titer > 1 : 100. The analysis of this variable according to the three ranges of disease severity showed similar behavior to that observed in RBD-specific titers (Figure 5(b)). On the other hand, the sera from convalescents with severe symptoms showed the highest levels of inhibitory antibodies blocking RBD-ACE2 binding, followed by patients with mild and moderate symptoms, while antibodies from asymptomatic convalescents displayed lower or null capacity to inhibit RBD-ACE2 interaction (Figure 5(c)).

**Figure 6:**
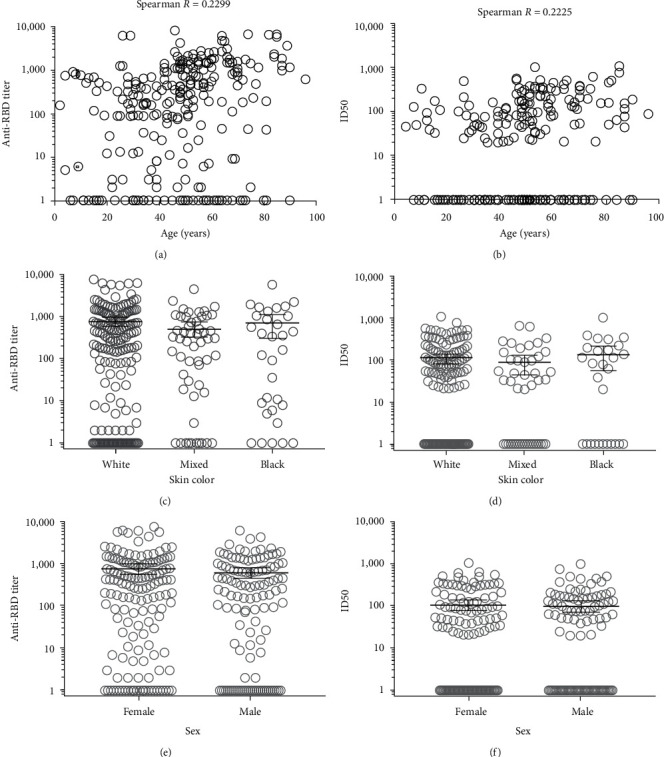
Impact of demographic features of Cuban COVID-19 recovered patients on the magnitude and quality of the anti-RBD antibody response. Anti-RBD IgG titer was determined by RBD ELISA. Serum samples were incubated on plates coated with the RBD-mFc and were detected with an anti-human IgG antibody conjugated to horseradish peroxidase (HRP). Half-maximal inhibitory dilution (ID50) was determined by surrogate virus neutralization test (sVNT). Inhibition of binding of RBD-mFc to hACE2-hFc was assessed after preincubation of RBD-mFc with sera samples. Spearman correlation was used to determine the association between the age of convalescents and (a) anti-RBD titer and (b) ID50. Stratified sera according to skin color (white, mixed, and black) or their sex (female and male) were tested by (c and e) RBD ELISA and (d and f) sVNT, respectively.

A similar approach was used to evaluate the impact of individual characteristics on the magnitude and quality of the RBD-specific immune response. None of the analyzed variables (age, skin color, and sex) correlated with humoral response (Figures 6(a), 6(b), 6(c), 6(d), 6(e), and 6(f)), which pointed to severity as the main determinant of the magnitude and quality of antiviral humoral response.

### 3.5. RBD-Specific IgG Antibodies Decline over Time, Albeit Their Inhibitory Capacity is Maintained

**Figure 7:**
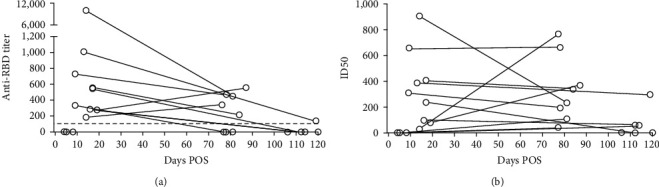
Longitudinal study of anti-RBD antibody response in COVID-19 recovered patients. Serum samples of 14 recovery patients were collected at t1 (3–17 days POS) and t2 (60–120 days POS). (a) Anti-RBD IgG titer was determined by RBD ELISA. Serum samples were incubated on plates coated with the RBD-mFc and were detected with an anti-human IgG conjugated to horseradish peroxidase (HRP). (b) Half-maximal inhibitory dilution (ID50) was determined by surrogate virus neutralization test (sVNT). Inhibition of binding of RBD-mFc to hACE2-hFc was assessed after pre-incubation of RBD-mFc with serum samples.

We further evaluated the dynamics of the humoral response over time using sequential serum samples (t1 and t2) from 14 COVID-19 convalescents. Most (*n* = 12/14) had mild or moderate symptoms. Anti-RBD antibodies were heterogeneous among individuals (t1 mean IgG titers, 1 : 994 [95% CI, 1 : 512 to 1 : 2,500] and t2 mean IgG titers, 1 : 155 [95% CI 1 : 33 to 1 : 277]; Figure 7(a)). Due to the small sample size, we were unable to carry out statistical analysis. The majority of participants (9/14) developed detectable levels of anti-RBD IgG during the end of the acute phase, and only the five participants which serum sample was collected the first 8 days of disease, had undetectable anti-RBD IgG. The anti-RBD titers gradually decreased by about sixfold during subsequent 4 months (from 3–17 to 60–120 days POS). At both times, the majority of serum samples achieved at least 50% neutralization by sVNT at a 1 : 25 dilution (t1: 10/14 and t2: 13/14). The longitudinal evaluation of quality of the antibody response suggested that their inhibitory capacity remains unchanged over time (Figure 7(b)).

## 4. Discussion

COVID-19 was declared as a pandemic in March 2020, a few months later from the first reports of SARS-CoV-2 infected patients [2]. Part of the efforts to contain this pandemic have been focused on studying the immune response generated by the viral infection. In this report, we evaluated, for the first time, the magnitude and functionality of the antibody response against RBD in Cuban COVID-19 convalescents.

Considering the novelty of the virus, it was necessary to develop new analytical tools and methodologies for testing antibodies in convalescents. Despite many serological approaches detect serum antibodies against the N and the S protein [11], we chose the RBD as viral antigen because this region is the main target of neutralizing antibodies [16] and is poorly conserved between different coronaviruses [17]. Although we assessed sera collected before the COVID-19 pandemic, which could presumably be positive from past common human CoV infections [18, 19], the discriminatory potency between prepandemic and convalescent sera of both ELISAs, highlights the specificity of the RBD as antigen [17]. Additionally, anti-RBD antibody titers from cohabitant cohort was significantly lower than that from convalescents. However, anti-RBD antibodies were detected in 15% (12/79) of cohabitant serum samples, which suggests that probably these participants were infected with SARS-CoV-2, but at the time of collecting nasopharyngeal swabs, the viral burden was low enough to be missed by RT-PCR. Our study, which included more than 270 serum samples from Cuban COVID-19 convalescents with varying levels of severity, strongly validates the use of RBD as the target for the specific antibodies evaluation.

A broad variety of recombinant versions of SARS-CoV-2 RBD have been produced for analytical purposes, using diverse expression systems [20, 21, 22, 23, 24]. These examples differ in the exact length of the RBD moiety itself, the design of additional fused protein domains or short tags for stabilization and purification, the choice of host cells (insect or mammalian), and the method of transfection (stable, semi-stable, or transient). Even though the RBD has been often defined as the sequence comprised between residues 319 and 541 of S protein [20, 21], slightly shorter versions have also been useful for detecting anti-RBD antibodies [17, 22]. The segment spanning from residue 328–533 was selected for the current study, because it contains the minimal compact folding unit where ACE2 binding determinants reside [14] plus short N- and C-terminal extensions and excludes the unpaired C538 which could give rise to nonnatural disulfide bond formation. Such RBD sequence was fused to a mouse IgG2a Fc region due to the usefulness of immunoglobulin Fc domains as fusion partners within a variety of recombinant proteins including RBD variants [23]. The presence of Fc moiety allowed purification by protein A affinity chromatography and sensitive detection using available secondary antibodies. Mouse origin of Fc ensured antigen compatibility with immunoassays aimed at detecting the presence and function of human antibodies. Expression levels of the RBD-mFc fusion protein were higher than the ones of His-tagged RBD also produced by HEK-293 cells [20].

Additionally, the good performance of our assays was confirmed because both tests showed a relatively good correlation with external validated methods, which have been used to quantify the antibody levels during vaccine development and clinical trials [25, 26]. Such results might become particularly important when taking into consideration the main limitations of the cVNT [15]. Our tests may be an effective alternative way of detecting antibodies and their inhibitory ability with acceptable performance in COVID-19 patients or convalescents.

Once stablished analytical assays, we studied the impact of clinical and individual characteristics on the magnitude and functionality of the humoral response. One of the most significant strengths of our study was the recruitment of convalescent across all Cuban provinces, who experienced a varied degrees of disease severity, ranging from no symptoms to severe illness.

In agreement with previous reports, RBD-specific antibodies, their avidity index and neutralizing capacity were heterogeneous among Cuban convalescent individuals and significantly higher in those convalescents who recovered from severe illness [8, 27]. One possible interpretation is that severe disease is more likely to start with a high viral load/antigen burden [28, 29], and consequently, results in a stronger antigen-driven antibody response [30]. However, for reasons that are not fully understood, this robust humoral response elicited against SARS-CoV-2 is unable to effectively control the disease progression. Alternatively, other authors suggest that the direct relationship between disease severity and magnitude of humoral response could be associated with a direct pathogenic role of antibodies. Indeed, antibody-mediated SARS-CoV-2 uptake by monocytes and macrophages triggers an inflammatory death, knowing as pyroptosis, and consequently, the release of potent inflammatory mediators [31]. In support of this idea, highly inflammatory milieu created in severe COVID-19 would promote the extrafollicular expansion of a naive-derived and low-mutation IgG1 population of antibody-secreting cells and the formation of pathogenic autoantibodies against the glomerular basement. Our interpretation is that the association between antibody levels and disease outcomes might be result of a sustained and dysregulated immune response, which does not clear the viral infection despite prolonged, elevated, and neutralizing antibody titers across multiple isotypes and affinities, rather than the pathological role of antibodies. Actually, specific antibodies induced by vaccination or SARS-CoV-2 infection provide protection against a subsequent infection [8, 32].

A relevant demographic variable on the COVID-19 progression to acute respiratory distress syndrome, the high morbidity and mortality rate, is the age of patients [33]. Older convalescents developed higher amount of RBD binding and neutralizing antibodies, as has been reported previously [34]. The most straightforward interpretation is that the magnitude of the humoral response is not determined by the age, but depends on the disease severity. Indeed, in a univariate analysis, age correlated with higher neutralizing avidity, however, did not reach significance in the multivariate linear regression [35].

Understanding the dynamics of humoral response in COVID-19 convalescents has important implications for predicting protection against reinfection. We obtained that the highest levels of RBD-specific antibodies were developed in the early convalescence phase, while still at the late, anti-RBD IgG titers were detected above the threshold, similar to report by Rodda et al. [18], but lower than initial recovery phase, which is in agreement with the results obtained by Turner et al. [36]. Our findings agree with the anti SARS-CoV-2 B cell expansion during mild infection. The induced extrafollicular response in which some B cells differentiate into short-lived plasma cells, leads an initial influx of antibodies that decline over time [37], followed by the expansion of RBD-specific long-lived bone marrow plasma cells [36]. The overall maintenance of the neutralizing activity at late as 120 days POS compensates the contraction in the magnitude of humoral response and suggests an improvement in the antibodies' quality, consistent with germinal center maturation [18].

Our study has certain limitations worth noting. The assays implemented for IgG titer and avidity evaluation were not fully validated limiting the extent of the obtained results. Additionally, we did not calculate the sample size a priori. Instead, it was limited by the voluntary participation of convalescents and the availability of testing capacity. Indeed, the first cross-sectional study, which enrolled 237 Cuban COVID-19 recovered patients, allowed us to characterize the specific humoral response elicited by SARS-CoV-2 infection during the first wave of the COVID-19 pandemic. However, the small size sample of the second study (14 convalescents, who is mainly restricted to individuals recovered from mildly symptomatic) limited us to only describing the results concerning the durability of the specific antibody response. Further studies should be conducted in order to conclude about antibody kinetics. Additionally, more sampling time points over 120 days period would provide a more accurate understanding of the kinetics of humoral response.

## 5. Conclusions

After the pandemic outbreak, unprecedented global efforts are being devoted to understanding the immune response to SARS-CoV-2. The study of the dynamics of anti-viral antibodies elicited by infection, such as antibody levels, their durability and their inhibitory capacity, in Cuban COVID-19 recovered patients, has been essential to improve the Cuban vaccines development and our clinical management of the pandemic. We found that the magnitude and quality of RBD humoral response elicited by SARS-CoV-2 infection depend on the disease severity, meanwhile the antibody titles decline over time. Importantly, the development of new tools and methodologies, for a novel viral disease in an emergency situation guaranteed the serological studies in more than 270 serum samples.

## Figures and Tables

**Table 1:** Clinical and demographic characteristics of Cuban COVID-19 convalescents.

Characteristic	Cuban COVID-19 recovered patients				
	First outbreak				Second outbreak
	Total study population (*n* = 273)	Asymptomatic (*n* = 79)	Mild/moderate (*n* = 131)	Severe (*n* = 63)	Total study population (*n* = 14)
Age, median (range)	49 (1–96)	45 (1–80)	48 (4–96)	59 (23–90)	55 (28–82)
Sex (*n*)					
Female	150	45	69	36	5
Male	121	34	62	25	9
Skin color (*n*)					
White	182	52	86	44	8
Mixed	54	15	27	12	3
Black	32	12	14	6	3

## Data Availability

The data will be available upon reasonable request.
